# The effect of orthodontic forces on calcitonin gene-related peptide (CGRP) expression in the human periodontal ligament and its relationship with the human dental pulp

**DOI:** 10.4317/jced.59975

**Published:** 2022-11-01

**Authors:** Javier Caviedes-Bucheli, Jaime O. Moreno, Morella Aranguren-Carrero, Sandra Buitrago-Rojas, Rossant Lopez-Matheus, Gerson Martinez-Corredor, Luis-Eduardo Díaz-Barrera, Hernan-Dario Muñoz-Alvear, Jose-Francisco Gomez-Sosa, Hugo-Roberto Munoz

**Affiliations:** 1 DDS, MSc, Professor & Researcher. Centro de Investigaciones Odontologicas (CIO), School of Dentistry, Pontificia Universidad Javeriana, Bogotá, Colombia; 2 DDS, PhD, Professor & Researcher. Postgraduate Department, School of Dentistry, Universidad Santo Tomas, Floridablanca Bucara¬manga, Santander, Colombia; 3 DDS. Postgraduate Department, School of Dentistry, Universidad Santo Tomas, Floridablanca Bucaramanga, Santander, Colombia; 4 DDS, MSc, Postgraduate Department, School of Dentistry, Universidad Santo Tomas, Floridablanca Bucaramanga, Santander, Colombia; 5 DDS. Postgraduate Department, School of Dentistry, Universidad Santo Tomas, Floridablanca Bucaramanga, Santander, Colombia; 6 DDS. Postgraduate Department, School of Dentistry, Universidad Santo Tomas, Floridablanca Bucaramanga, Santander, Colombia; 7 PhD, Professor & Researcher. Engineer School, Universidad de la Sabana, Chía, Colombia; 8 DDS. Endodontics Department, Universidad Cooperativa de Colombia, Pasto, Colombia; 9 DDS, PhD, Professor & Researcher. Unidad de Terapia Celular, Centro de Medicina Regenerativa, Instituto Venezolano de Investigaciones Cientificas, Caracas, Venezuela; 10 DDS, MSc, Professor & Researcher. Postgraduate Endodontics Department, School of Dentistry, Universidad de San Carlos de Guatemala, Guatemala

## Abstract

**Background:**

The purpose of this study was to quantify the effect of moderate and severe orthodontic forces on Calcitonin gene-related peptide (CGRP) expression in the healthy human periodontal ligament (PDL) and its possible relationship with the human dental pulp.

**Material and Methods:**

Ninety human periodontal ligament samples were obtained from healthy premolars where extraction was indicated for orthodontic reasons. Prior to extraction, teeth were divided in 3 groups of 30 samples each: I) Untreated teeth control group; II) Moderate force group: A 56 g force was applied to the premolars for 24 hours; and III) Severe force group: A 224 g force was applied to the premolars for 7 days. All periodontal ligament samples were processed and CGRP was measured by radioimmunoassay.

**Results:**

Greater CGRP expression was found in the severe force group, followed by the moderate force group. The lower CGRP values were for the untreated teeth. Kruskal-Wallis test showed statistically significant differences between groups (*p*<0.001). LSD post hoc tests showed statistically significant differences in CGRP expression between the untreated teeth and the severe forces group (*p*<0.001). Differences between the moderate and severe force groups were statistically significant (*p*<0.001). There was no statistically significant differences between the untreated teeth and the moderate forces group (*p*<0.261).

**Conclusions:**

CGRP expression in human periodontal ligament increases when teeth are submitted to severe orthodontic forces. This elevated expression of CGRP, which is proportional to the applied force, may affect the way the dental pulp responds to different stimuli from the orthodontic forces.

** Key words:**Calcitonin gene-related peptide, orthodontic force, human periodontal ligament, neurogenic inflammation.

## Introduction

The periodontal ligament (PDL) is a specialized, highly vascularized and innervated fibrous connective tissue (1). Its main functions are attaching the tooth to the alveolus and protecting the tooth against excessive masticatory pressure (2). It also plays a role in the physiological and therapeutic tooth movement and in repairing the periodontium when this tissue has been damaged by dental trauma or orthodontic movements (3).

The PDL is directly related to and in contact with the dental pulp through the dentinal tubules located in throughout the root (4). Similarly, the accessory or lateral canals contain connective tissue and blood vessels that communicate the canal system with the PDL, being permeable to inflammatory mediators. Although it is well known that the main route of connection between the pulp, PDL and periapex is through the apical foramen (5). Due to this close relationship, when the pulp or the PDL respond to stimuli such as orthodontic movements, they generate an inflammatory neural and vascular response mediated by neuropeptides such as calcitonin gene related peptide (CGRP) in both tissues (6).

Orthodontic forces lead to considerable changes to the cell population of the PDL and dental pulp, as well as in their vascular and nervous structures (7). It has been shown that the remodeling response of PDL to orthodontics is influenced by the stress level and the inflammation generated in the tissue, which are directly related to duration, type and magnitude of the force (8).

PDL sensorial nerve fibers play a significant role in modulating the inflammatory reaction through the release of neuropeptides, including CGRP. This peptide is capable of triggering vasodilation, plasma extravasation, immune system activation, chemotaxis, recruitment and/or regulation of inflammatory cells such as macrophages, mast cells and lymphocytes (9,10). This neurogenic inflammation process activates local mechanisms (such as vascular stasis, hyalinization and tissue necrosis) that could lead to root resorption (11-13).

Previous studies have associated extrusive orthodontic forces with some deleterious effects in dental pulp and PDL, ranging from vascular stasis to root resorption (6,13-15). Different range forces have been recommended in adults to prevent deleterious effects on these tissues, showing that the optimal range lies between 50 to 100 cN (25-50 gr). However, the 100 to 150 cN (50-75 gr) range has also been suggested. Orthodontic forces range between 150-200 cN (75-100 gr) and higher values are capable of generating pulp and PDL damage (6,16).

Quantification of CGRP in human PDL will provide scientific knowledge that could help explain the biological mechanisms involved during periapical response to orthodontic movement, it possible relationship with the human dental pulp. Therefore, the purpose of this study was to determine the expression of CGRP in human PDL of teeth subjected to moderate and severe orthodontic forces.

## Material and Methods

Patients participating in the study were 18-27 years old, healthy, not medicated and nonsmoking with premolars extraction indicated for orthodontic reasons. All teeth used were caries- and restoration-free with complete root development determined radiographically (and confirmed visually after extraction), without signs of periodontal disease or traumatic occlusion and without previous orthodontic forces.

-Experimental Procedure

PDL samples were obtained from 90 premolars that were randomly divided into 3 groups of 30 premolars each, as follows: Untreated teeth control group; Moderate force group; Severe force group. Teeth in moderate and severe orthodontic force groups were submitted to tipping and extrusion orthodontic movements.

Orthodontic forces were applied following the exact same methodology that was used in a previous study, in which, prior to orthodontic force application, the occlusal surface of the first mandibular molar was raised with a block of resin (Filtek Z350, 3M Espe, Seefeld, Germany) until the premolars were out of occlusion. A convertible standard buccal tube (OrthoOrganizer, Carlsbad, CA) was bonded over the buccal face of the first molar with resin (Light Bond, Reliance Orthodontic Products Inc, Itasca, IL). A MBT slot size 0.022 bracket (Ref. 702-393 MC, OrthoOrganizer) was bonded over the buccal face of the premolars. One 0.0017 x 0.025 in titanium molybdenum alloy (TMA) wire cantilever was inserted into each first molar tube and the wire was bent buccally to form a helix (6).

The cantilever was clinched to the distal end of the tube and exerted a tipping and extrusive force on the premolar. For the teeth in the moderate force group, the activation angle was 45º with a force of 56g. For the severe force group, the activation angle was 90º with a force of 224g. For both groups, forces were measured with an orthodontic dynamometer. Once the force was measured, the free-end of the sectional arch was hooked to the bracket with a metallic ligature. Seven days after, the ligature, the sectional arch, the tube and the resin block were removed in order to perform the extraction procedure.

All teeth were anesthetized with 1.8 mL 4% prilocaine without vasoconstrictor by infiltrative injection for upper premolars and inferior alveolar nerve block injection for lower premolars.

-Sample Collection

Teeth in the control and orthodontic forces group were extracted 10 min after anesthetic application with conventional methods and without excessive injury to PDL. Immediately after extraction, PDL samples were obtained from the entire length or the root with a 5/6 Gracey’s periodontal curette, placed on a 0.5ml Eppendorf tube, snap-frozen in liquid nitrogen until use.

-Radioimmunoassay (RIA)

PDL samples were defrosted without thermal shock, dried on a filter and individually weighed on an analytical balance. Neuropeptide was extracted by adding 150 µL of 0.5 mol L-1 acetic acid and double boiling in a thermostat bath for 30 min in accordance with previously reported protocols (6,12,17-20).

CGRP expression was determined by competition binding assays using a human CGRP-RIA kit from Phoenix Peptide Pharmaceutical (Ref. RK-015-02, Belmont, CA). Fifty µL of each sample solution were incubated in polypropylene tubes at room temperature for 20 h with 100 µL of primary antibody and 100 µL of different CGRP concentrations (10 pg mL-1 –1280 pg mL-1). Then, 50 µL of 125I-CGRP was added and left incubate for another 24 h. Bound fractions were precipitated by the addition of 100 µL of a secondary antibody (Goat Anti- Rabbit IgG serum), 100 µL of normal rabbit serum and 500 µL of RIA buffer containing 1% polyethylene glycol 4000. After 2 h of incubation at room temperature, tubes were spun at 3000 rpm for 45 min at 4ºC. The supernatants were decanted, and pellet radioactivity was read on a Gamma Counter (Gamma Assay LS 5500; Beckman, Fullerton, CA). Standard curves of authentic peptide were made in buffers identical to the tissue extracts on semi log graph paper.

-Statistical Analysis

Values are presented as CGRP concentration in pmol per mg of PDL. Kruskal Wallis test was performed to establish statistically significant differences between groups (*P*<.05). LSD post-hoc comparisons were also performed.

## Results

CGRP was found to be present in all PDL samples (Table 1). Highest CGRP levels were observed in the severe force group, with a mean value of 1.1608 ± 0.2937 (median 1.1384) pmol CGRP per mg of PDL, followed by the moderate force group with a mean value of 0.0721 ± 0.0122 (median 0.0717) pmol CGRP per mg of PDL. Lowest CGRP levels were observed in the untreated teeth control group samples with a mean value of 0.0225 ± 0.0067 (median 0.0220) pmol CGRP per mg of dental pulp.

**Table 1:**
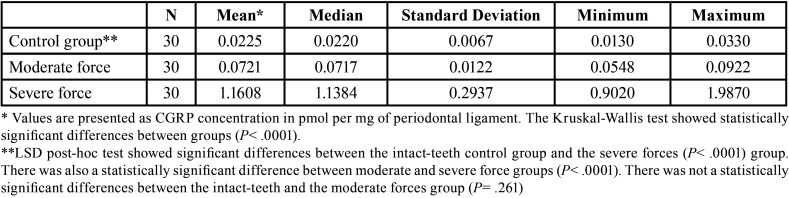
CGRP expression in periodontal ligament from healthy human premolars after moderate and severe orthodontic force application.

Kruskal-Wallis test showed statistically significant differences between groups (*P*<0.0001). LSD post hoc tests showed significant statistical differences between the untreated teeth control group and the severe force group (*P*<0.0001). Differences between the moderate and severe force groups were also statistically significant (*P*<0.0001). There was no statistically significant differences between the untreated teeth and the moderate force groups (*P*= 0.261).

## Discussion

Current evidence suggests that orthodontic movements generate inflammatory alterations in the PDL and dental pulp that are directly correlated with the magnitude, direction and duration of the force applied (2,16). CGRP released from C-fibers upon stimulation by mechanical stress, is capable of regulating the inflammatory process, by controlling vascular tone and blood flow, leading to rapid and large arrival of immune system cells and inflammatory mediators (9,13). During this process, pulp and PDL microcirculation undergoes dynamic changes that limit their ability to remove metabolic waste products and to maintain a harmonic interstitial pressure. In consequence, tissue edema takes place (13,21,22).

Prior to extraction, premolars in experimental groups were submitted to 7 days of moderate or severe orthodontic force application. Both experimental groups used a cantilever made of 0.017 x 0.025 TMA wire. This wire is adequate for the experiment, due to its rectangular shape and its long transverse diameter that allows proper bucco-lingual control, with an adequate elasticity module (23). In the present research, it was determined that activation angles of 45º and 90º generate 56- and 224-g forces respectively, as measured with a dynamometer.

Local anesthetic used in all groups of this study was 4% prilocaine without vasoconstrictor to prevent neuropeptide expression becoming attenuated by vasoconstrictors as previously demonstrated (24).

Previous studies have investigated the effect of different mechanical stimuli over PDL, including orthodontic movement, occlusal trauma and root canal preparation (11,12). These studies concluded that CGRP-containing nerve fibers play an important role in regulating blood flow, remodeling tissue, and modulating pain and tooth movement during orthodontic treatment (25). It also has been reported that CGRP amplifies the inflammatory effects of Substance *P* by increasing the release of inflammatory mediators and thus, perpetuating this process leading to root resorption (9,26).

A significant increase in PDL CGRP levels after orthodontic movement supports the hypothesis that sensory C-type nerve fibers of PDL, as well as in pulp, respond to the application of orthodontic forces releasing neuropeptides which can provoke an alteration in tissue homeostasis and pain sensitivity by triggering the release of inflammatory mediators (6,11). If the orthodontic force remains constant for longer periods of time, nerve fibers could become sensitized enhancing pain response and favoring root resorption (12,27), due to hyalinization and necrosis of PDL nerve fibers as a result of a disorganization in the collagen matrix and damage to nervous terminals (28). Consequently, if the inflammatory process is maintained in the PDL, the increase in inflammatory mediators and defense cells such as macrophages, mast cells, and lymphocytes can induce an inflammatory process in the pulp (6).

There was a non-significant increase of PDL CGRP levels after moderate orthodontic forces application. This type of force could be associated to less aggressive and temporary damage to the PDL, due to a minor alteration in blood flow and collateral microcirculation caused by the inflammatory process, which could be a biological explanation of the pain response after the activation of orthodontic forces that comes back to normal in a few days without generating permanent damage to PDL (11,12), In the same way, the pulp will undergo a mild reversible inflammatory effect consistent with the increase in blood flow due to the CGRP that migrates to the pulp through dentinal tubules, lateral ducts or apical foramen to the PDL. However, there are no irreversible changes in the vascular structure of the pulp and if they occur, they are rapidly compensated through angiogenesis (29,30).

**Figure 1:**
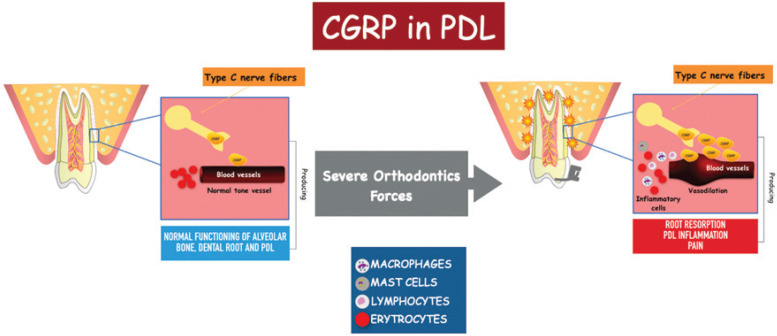
Summary of the possible biological response to the increase CGRP levels in PDL, where C-type nerve fibers release CGRP which causes vasodilation, release of inflammatory cells that could produce PDL inflammation, root resorption and pain when severe orthodontics forces are applied to the tooth.

The teeth submitted to severe orthodontic forces showed a significant increase in PDL CGRP levels. This could be correlated with a greater damage to PDL, and consequently to dental pulp, leading to aggressive root resorption in the short and long terms (6,31). CGRP is capable to activate both osteoclast and osteoblast cells, regulating the resorptive process to compensate the trauma caused by orthodontic movement (9,12). The higher expression of CGRP levels in both PDL and pulp after severe orthodontic forces could also be explained due to similarity in the vascular, cellular and immune responses of both tissues (12), Regarding the pulp, the increase in CGRP acts synergistically with the expression of SP, intervening in the formation of new vessels, stimulating endothelial cells through the cAMP-PKA pathway, in addition to increasing the number of collagen fibers followed by mineralization of the tissue (30,32). Sustained expression of CGRP will produce fibrosis of the tissue due to hyalinization and reduction of blood vessels replaced by mineralized tissue, thus an irreversible state will be produced in the pulp, reaching necrosis, and therefore, magnifying the inflammatory response of PDL and accentuating resorption processes (Fig. 1) (6,12,29,33).

CGRP release is immediate and sustained, leading to greater degenerative damage when the forces are applied for longer periods of time. Clinically, this situation could be observed in some orthodontic treatment patients that suffer occlusal trauma due to the alterations in their occlusion generated during orthodontic movement, magnifying the inflammatory process both in pulp as in PDL, and leading to negative consequences ranging from pain to aggressive root resorption, depending on the duration and magnitude of the stimulus (12).

The 7 days period frame evaluated in the present study has been proved to be enough to allow the neuropeptide release from nervous terminals prior to being degraded by endogenous peptidases, as CGRP release is immediate, calcium-dependent and of short-term (9). Although some possible mechanisms for extracellular CGRP increased release have been proposed (34), including: (i) increased synthesis of the neuropeptide in the trigeminal ganglia; (ii) increased rate of transport; and (iii) decreased levels of peptidases; more recent evidence has demonstrated that mRNA transcripts are transported to peripheral terminals, suggesting that peptide synthesis could occur directly in the peripheral terminals (10).

Finally, it is also important to point out the clinical relevance of using moderate and intermittent orthodontic forces, which are capable of generating an adequate tooth movement, limiting the damage and allowing PDL and pulp to recover from the injury (11).

## Conclusions

CGRP expression in the human periodontal ligament increases when teeth are subjected to severe orthodontic forces. This elevated expression of CGRP, which is proportional to the applied force, may affect the way the dental pulp responds to different stimuli from the orthodontic forces.
